# Formalin Itch Test: Low-Dose Formalin Induces Histamine-Independent, TRPA1-Mediated Itch in Mice

**DOI:** 10.3389/fmed.2021.627725

**Published:** 2021-02-15

**Authors:** Xu Liu, Jiang-Tao Zhang, Yue Hu, Wen-Qi Shan, Zhi-Hong Wang, Qing-Yue Fu, Dan-Ni Fu, Jiang Ji, Tong Liu

**Affiliations:** ^1^Department of Dermatology, The Second Affiliated Hospital of Soochow University, Suzhou, China; ^2^Jiangsu Key Laboratory of Neuropsychiatric Diseases and Institute of Neuroscience, Soochow University, Suzhou, China; ^3^Institute of Pain Medicine and Special Environmental Medicine, Nantong University, Nantong, China; ^4^College of Life Sciences, Yanan University, Yanan, China

**Keywords:** itch, pain, formalin, TRPA1, dorsal root ganglion

## Abstract

Chronic itch is a common distressing symptom of many diseases, which reduced patient's quality of life. The mechanistic study on itch and screening for new anti-itch drugs require the development of new pre-clinical itch animal models. Herein, we established an acute itch model by intradermal (i.d.) injection of low-dose formalin into the neck or cheek in mice. In mice, i.d. injection of formalin (0.1–5%) in the nape of the neck evoked robust scratching behavior in a dose-dependent manner and the dose–response curves showed an inverted “U” shape. I.d. injection of formalin (0.3–0.6%) into the cheek evoked scratching in mice but wiping in rats, while formalin (1.25–5%) induced mixed wiping and scratching behavior in both mice and rats. Further, we found that 0.3% formalin-induced scratching was histamine-independent and significantly attenuated by transient receptor potential ion channel A1 (TRPA1) inhibitor (HC030031) or in TRPA1 knockout (KO) mice, but not affected by transient receptor potential ion channel V1 (TRPV1) inhibitor (capsazepine) or in TRPV1 KO mice. Additionally, 0.3% formalin-induced up-regulation of phosphorylation of extracellular regulated protein kinases (p-ERK) in the dorsal root ganglion (DRG) and scratching were suppressed by intrathecal injection of MEK inhibitor U0126 in mice. Incubation of 0.03% formalin induced the accumulation of intracellular reactive oxygen species (ROS) in the cultured DRG-derived cell line ND7-23, and formalin-induced itch was suppressed by antioxidants in mice. Finally, perfusion of 0.03% formalin induced elevation of intracellular calcium in a subset of primary cultured DRG neurons of mice. Thus, these results indicate that low-dose formalin induced non-histaminergic itch by activation of TRPA1 in mice, which may be employed as a useful acute itch model for screening potential anti-itch drugs.

## Introduction

Itch (pruritus) is defined as a common unpleasant sensation that causes desire or reflex to scratch (1, 2). Itch can be divided into acute itch and chronic itch (3). Acute itch may have a protective function for the body in order to remove potential harmful substances by scratching behavior (4). However, chronic itch is a common symptom in the complications of skin diseases (e.g., atopic dermatitis and psoriasis) (5, 6), metabolic diseases (e.g., diabetes) (7), liver diseases (e.g., cholestasis) (8), kidney diseases (e.g., uremic pruritus) (9), and seriously affecting patient's quality of life. Based on mechanisms, itch can be further divided into histamine-dependent itch and histamine-independent itch (10). Generally, histamine-dependent itch (such as allergy itch) is mediated by the histamine receptor H1 and H4, which is clinically treated with antihistamine drugs (11). However, chronic itch is often resistant for the treatment of antihistamines (3), which suggests histamine-independent mechanisms involved. Thus, to elucidate the mechanisms of histamine-independent itch and to screen new anti-itch compounds, it is urgently needed to develop new pre-clinical itch animal models (12).

Traditionally, formalin test has been long-term used for studying the underlying mechanism of acute inflammatory pain and screening potential analgesics in rodents (13, 14). In the past decades, it was found that formalin is able to directly activate several ion channels that are involved in the generation of pain, including transient receptor potential ion channel A1 (TRPA1) (15), transient receptor potential ion channel V1 (TRPV1) (16), or transient receptor potential ion channel V4 (TRPV4) (17). Recently, it was demonstrated that several TRP channels, including TRPV1, TRPA1, and TRPV4, are important mediators for both acute and chronic itch (18, 19). For example, TRPV1 was demonstrated to mediate histamine-dependent itch (20). TRPA1 plays a critical role in the genesis of histamine-independent itch, such as Mas-related G protein-coupled receptors (Mrgprs)-mediated itch (21, 22), oxidative stress-induced itch (23, 24), endothelin-induced itch (25), and 5-HT_7_ receptor-mediated itch (26). In addition, it was demonstrated that TRPA1 also contributed to the pathogenesis of chronic itch, including dry skin-induced itch (27), bile acids TGR5-mediated cholestatic itch (28), tacrolimus-induced contact dermatitis pruritus (29), and imiquimod-induced psoriatic itch (30, 31). In addition, TRPV4 has also been shown to contribute to serotonin-induced itch and chronic allergic itch in mice (32, 33). Interestingly, several studies have provided several important clues that show that formalin-induced behavioral responses may have some itch component (34, 35). However, whether and how formalin induces itch is still unclear.

In the present study, we found that intradermal (i.d.) injection of low-dose formalin (0.3%) was able to evoke obvious scratching behavior in both neck and cheek models of mice, in a histamine-independent manner. Furthermore, the activation of TRPA1, oxidative stress, and extracellular regulated protein kinase (ERK) signaling were involved in low-dose formalin-induced itch in mice. Together, our results indicated low-dose formalin-induced histamine-independent itch in mice, and this new “formalin itch test” may be used for screening potential novel anti-itch compounds, especially for histamine-independent itch.

## Materials and Methods

### Animals

Male ICR mice, C57BL/6J mice, and Sprague–Dawley (SD) rats (6–8 weeks old) were obtained from the Shanghai SLAC Laboratory Animal CO., LTD. (Shanghai, China). Male *Trpa1*^−/−^and *Trpv1*^−/−^ mice were purchased from Jackson Laboratories (Bar Harbor, ME, USA). *Trpv4*^−/−^ mice were produced by Cam-Su Genomic Resource Center, Soochow University. All animals were kept on a 12-h light/dark cycle with free access to food and water, and the rooms were maintained at 22 ± 2°C and 40–60% humidity. All animal experiments were conducted in accordance with the National Institutes of Health Guide for the Care and Use of Laboratory Animals and the guidelines of the International Association for the Study of Pain.

### Neck Model of Acute Itch

As previously reported (36, 37), mice or rats were shaved at the nape of the neck more than 2 days before experiments. On the day of behavioral testing, mice or rats were placed in separate small plastic chambers (for mice: 10 × 10 × 12.5 cm^3^; for rats: 20 × 20 × 25 cm^3^) on an elevated metal mesh floor at least 30 min for habituation. Under brief anesthesia with isoflurane, 50 μl of compound 48/80 (100 μg), chloroquine (CQ, 200 μg), formalin (0.03–5%), and allyl isothiocyanate (AITC, 10–400 μg) were injected i.d. into the neck of mice through a 26G needle. Rats were injected with 100 μl of formalin (0.03–5%) into the nape of the neck. Immediately after the injection, mice were returned to the chambers and video-recorded for 30 min (Sony HDRCX610, Shanghai, China). The video was then played back offline and scratching behavior was quantified in a blinded manner. Scratching behavior occurred when mice lifted their hindpaws to scratch shaved skin and returned the paws to the floor or to their mouths. The drugs are different doses of formalin (0.03, 0.3, 0.6, 1.25, 2.5, and 5%) and different doses of AITC (10, 50, 100, 200, and 400).

### Cheek Model

As previously reported (38), mice or rats were shaved at the cheek more than 2 days prior to experiments. One day after shaving, the animals were moved to small plastic chambers (for mice: 10 × 10 × 12.5 cm^3^; for rats: 20 × 20 × 25 cm^3^) on an elevated metal mesh floor and allowed to acclimate for at least 30 min. Under brief anesthesia with isoflurane, mice or rats were given an i.d. injection of drugs into the cheek (for mice: 20 μl; for rats: 25 μl). After injection, the mice were immediately returned to the chambers and recorded for 30 min (Sony HDRCX610, Shanghai, China). The video was subsequently played back offline; scratching behavior and wiping behaviors were quantified in a blinded manner. Count scratch bouts and wiping behaviors, respectively. The wiping behavior means that mice or rats raise a forelimb toward the cheek times over 1 s or a few seconds, then put their forelimb down. This series of actions were counted as one wiping. Formalin (0.03, 0.3, 0.6, 1.25, 2.5, and 5%) and AITC (10, 50, 100, 200, and 400 μg) were used.

### Formalin Test

As previously reported (13), mice were acclimated to the environment (small plastic chambers 10 × 10 × 12.5 cm^3^) 1 h before the behavioral testing. Mice were given an intraperitoneal (i.p.) injection of morphine (3 mg/kg in saline) or saline. After 30 min, mice were given an intraplantar injection of 20 μl of 5 or 0.3% formalin in the right hindpaw. The behaviors of the animals were observed for 45 min to evaluate the total time each animal spent in lifting, licking, shaking, or biting their injected hindlimb. Nociceptive behavior was video-recorded (Sony HDRCX610, Shanghai, China) at 45° below the observation chamber. Analysis of the acute phase (0–10 min; phase 1) and the inflammatory phase (10–45 min; phase 2) was performed by experimenters that were blinded to the treatments.

### Western Blotting

Mice were deeply anesthetized with isoflurane and underwent cardiac perfusion with normal saline, after i.d. injection of 0.3 and 5% formalin in the neck of mice for 10 and 30 min. The dorsal root ganglion (DRG) of the C1–C8 segments were obtained and homogenized in a RIPA buffer containing a mixture of phosphatase inhibitors and protease inhibitors. The protein concentration in the RIPA buffer was measured by Pierce bicinchoninic acid (BCA) protein assay (Thermo), and sodium dodecyl-sulfate polyacrylamide gel electrophoresis (SDS-PAGE) sample loading buffer was added into the RIPA buffer, and the proteins were separated by SDS-PAGE. After transfer, the blots were blocked with 5% non-fat milk in Tris–HCl Buffer Saline (TBS) for 1 h at room temperature and the PVDF membranes were incubated overnight at 4°C with primary monoclonal anti-p-ERK (mouse, 1:1000; Santa Cruz Biotechnology, CA) or primary monoclonal anti-ERK (mouse, 1:1000, Vazyme, Nanjing, China). The blots were washed and incubated with horseradish peroxidase-conjugated goat anti-mouse IgG secondary antibody (1:2000, Vazyme). Protein bands were visualized using an enhanced chemiluminescence detection kit (Pierce), and the band densities were assessed and analyzed with NIH ImageJ software (NIH, Bethesda, MD).

### Measurement of Intracellular Reactive Oxygen Species in ND7-23 Cells

ROS level was detected by the DCFDA/H2DCFDA–cellular ROS assay kit (ab113852). The ND7-23 cells were cultured in the six-well plate up to 60–70%, and 0.03% formalin was added in the plate after pre-treatment with NAC (100 μmol/L) for 15 min and then incubating at 37°C for 30 min. The medium was removed and cells were stained with DCFH-DA (25 μmol/L) and incubated at 37°C for 30 min. The cells were washed times later and transferred to 1.5-ml tubes for flow cytometry (FC500; Beckman Coulter, Brea, CA). Similarly, DRG neurons were stained with DCFH-DA, and the AXIO SCOPE A1 was used to take images. Finally, fluorescence intensity was analyzed by ImageJ.

### Dorsal Root Ganglion Neuron Culture, Calcium Imaging, and Analysis

Extirpated cervical DRGs of neonatal mouse were dissociated by incubation for 30 min at 37°C in a culture medium (Neurobasal with 10% fetal bovine serum and 1% penicillin–streptomycin) containing 0.2% Collagenase D (Roche) followed by a 9-min incubation in 4 ml of culture media with 0.125% Trypsin–EDTA (NCM). Cells were fully dissociated with a pipette and filtered with a 70-μm cell strainer (NEST). Dissociated neurons were seeded on poly-L-lysine/laminin (Sigma) microscope cover glass (NEST). The neurons were incubated in an incubator (Thermo Fisher Scientific) humidified at 37°C, with 5% CO_2_. Culture media were supplemented with 2% B-27. For Ca^2+^ imaging experiments, primary cultured DRG neurons were loaded with 1 μg/ml Fura-2 AM (1:1000, Thermo Fisher) and 0.01% F-127 (w/v; Invitrogen) for 30 min in the dark at 37°C and perfusion DRG neurons with calcium imaging buffer (CIB) (130 mM NaCl, 5.6 mM KCl, 2.6 mM CaCl_2_, 1.2 mM MgCl_2_, 10 mM Hepes, and 5.6 mM D-glucose at pH 7.4). In chambers equipped with a custom four-channel perfusion valve control system, neurons were incubated with 0.03% formalin for 1 min and then infused with CIB to baseline, then with 0.06% formalin again for 1 min and infused with CIB to baseline, and finally with 56 mM KCl for 1 min and infused with CIB to baseline. To monitor changes in intracellular [Ca^2+^] with fluorescence images that were acquired using a Nikon Eclipse Ti microscope, emission at 510 nm was monitored from excitation at both 340 nm and 380 nm.

### Drugs and Administration

Formalin, compound 48/80 (Cat#C2313), CQ (Cat#C6628), Loratadine, N-acetyl-L-cysteine (NAC, Cat#A7250), N-tert-butyl-a-phenylnitrone (PBN, Cat#B7263), and AITC (Cat#377430) were obtained from Sigma-Aldrich (St. Louis, MO). Nalfurafine was purchased from MCE. HC-030031 (Cat#2896), Capsazepine (CPZ, Cat#0464), HC067047 (Cat#4100), and U0126 (Cat#U120) were obtained from Tocris (Bristol, UK). Morphine hydrochloride was obtained from China Northeast Pharmaceutical Group Shenyang No.1 Pharmaceutical CO., Ltd (Shenyang City, Liaoning Province, China). Naloxone hydrochloride was obtained from China Sinopharm Group Guorui Pharmaceutical CO., Ltd. (Huainan City, Anhui Province, China). PBN, capsazepine, and HC030031 were dissolved in 10% DMSO. Other reagents were dissolved in sterile saline unless specified otherwise. Formalin was dissolved in CIB for Ca^2+^ imaging analysis.

### Statistical Analysis

Data were analyzed using GraphPad Prism 6.1 (GraphPad, La Jolla, CA). All data were expressed as the mean ± standard error of the mean (SEM). Two-tailed Student's *t*-test was used for two-group comparisons. One-way ANOVA followed by *post-hoc* Bonferroni test was used for multiple comparisons. Two-way ANOVA followed by *post-hoc* Bonferroni test was used to analyze the data with repeated measures over a time course. Difference with *P* < 0.05 was considered to be statistically significant.

## Results

### Low-Dose Formalin Induces Itch Behavior in Mice, but Not in Rats

**Figure 1:**
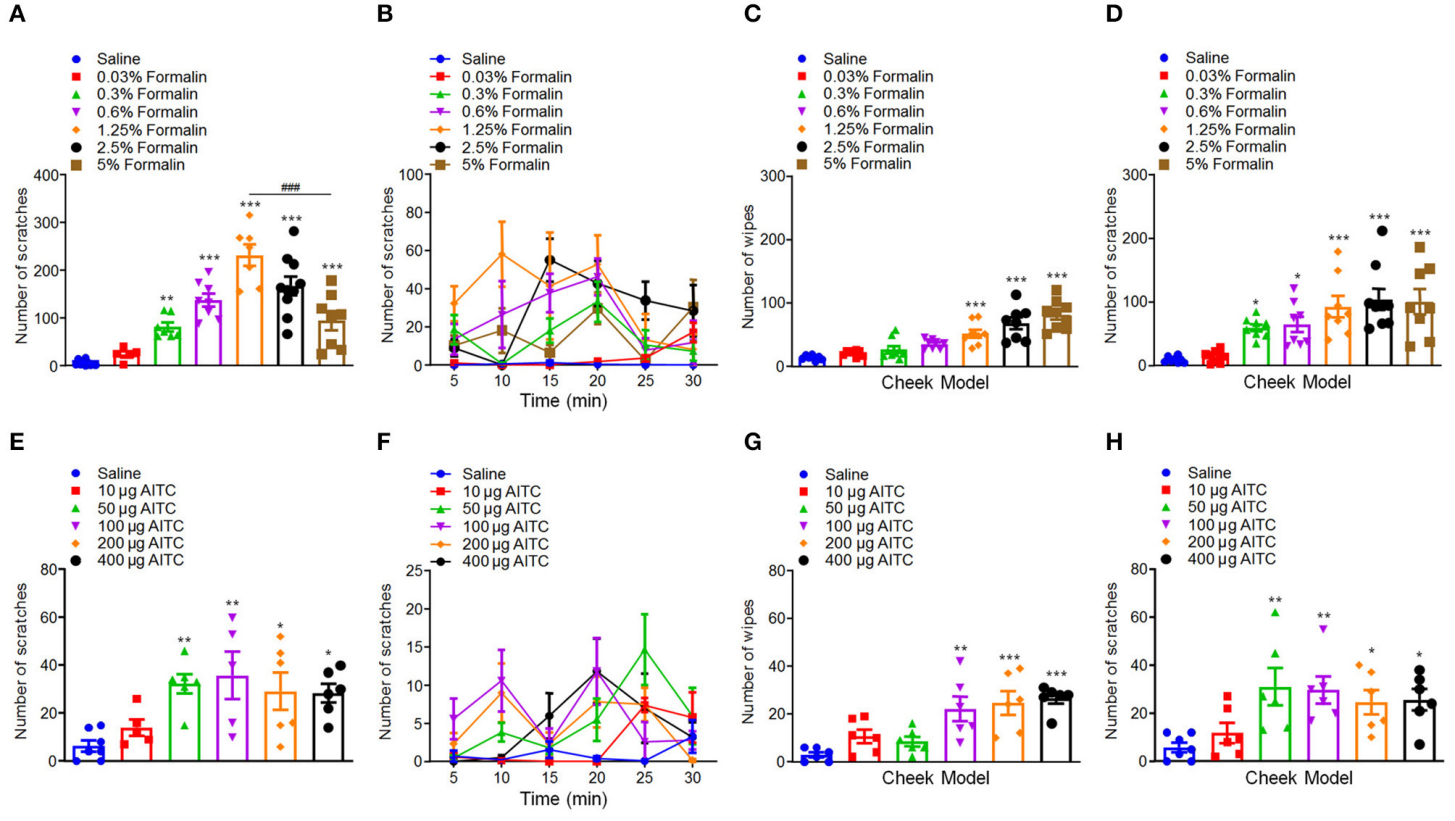
Low-dose formalin evoked scratching behavior in the neck and cheek models of mice. **(A,B)** The total number **(A)** and time course **(B)** of scratching behavior induced by intradermal (i.d.) injection of the different doses of formalin (0.03–5%) in the nape of the neck in mice. **(C,D)** The total number of wiping **(C)** and scratching behavior **(D)** induced by i.d. injection of the different doses of formalin (0.03–5%) in the cheek in mice. **(E,F)** The total number **(E)** and time course **(F)** of scratching behavior induced by i.d. injection of the different doses of AITC (10–400 μg) in the nape of the neck in mice. **(G,H)** The total number of wiping **(G)** and scratching behavior **(H)** induced by i.d. injection of the different doses of AITC (10–400 μg) in the cheek in mice (*P < 0.05, **P < 0.01, ***P < 0.001 vs. Saline, ^###^*P* < 0.001 vs. 1.25% Formalin, one-way AVOVA following *post-hoc* Bonferroni's test and unpaired Student's *t-*test; *n* = 5–10). All data are expressed by means ± SEM.

First, we investigated whether administration of formalin is able to induce itch in rodent or not. We found that i.d. injection of the different doses of formalin (0.03–5% in 50 μl) in the nape of the neck of mice were able to evoke scratching behavior in a dose-dependent manner [*F*_(6, 48)_ = 25.76, *P* < 0.0001; Figures 1A,B]. Formalin began to evoke scratching at 0.3% and research a peak at the dosage of 1.25%. However, the highest dose of formalin (5%) induced significantly less scratches than that of 1.25% formalin (*t*_13_ = 4.601, *P* = 0.0005; Figure 1A), which suggested an inverted “U” shape for the dose–response curve. The cheek model showed that the low-dose formalin (0.3%) only induces itch-indicative scratching but not pain-indicative wiping. However, the higher doses of formalin (1.25–5%) induced both wiping [*F*_(6, 51)_ = 19.81, *P* < 0.0001; Figure 1C] and scratching behaviors in mice [*F*_(6, 51)_ = 9.737, *P* < 0.0001; Figure 1D]. In addition, i.d. injection of a TRPA1 selective agonist AITC (10–400 μg) into the nape of the neck also evoked scratching behavior in a dose-dependent manner in mice [*F*_(5, 29)_ = 4.529, *P* = 0.0036; Figures 1E,F]. For the cheek model, it was demonstrated that low-dose AITC (50 μg) only induces itch-indicative scratching but not pain-indicative wiping, while the higher doses of AITC (100–400 μg) induce both wiping [*F*_(5, 30)_ = 8.286, *P* < 0.0001; Figure 1G] and scratching [*F*_(5, 31)_ = 4.335, *P* = 0.0042; Figure 1H]. Thus, the results indicated that the low-dose formalin is able to induce acute itch behavior in mice.

**Figure 2:**
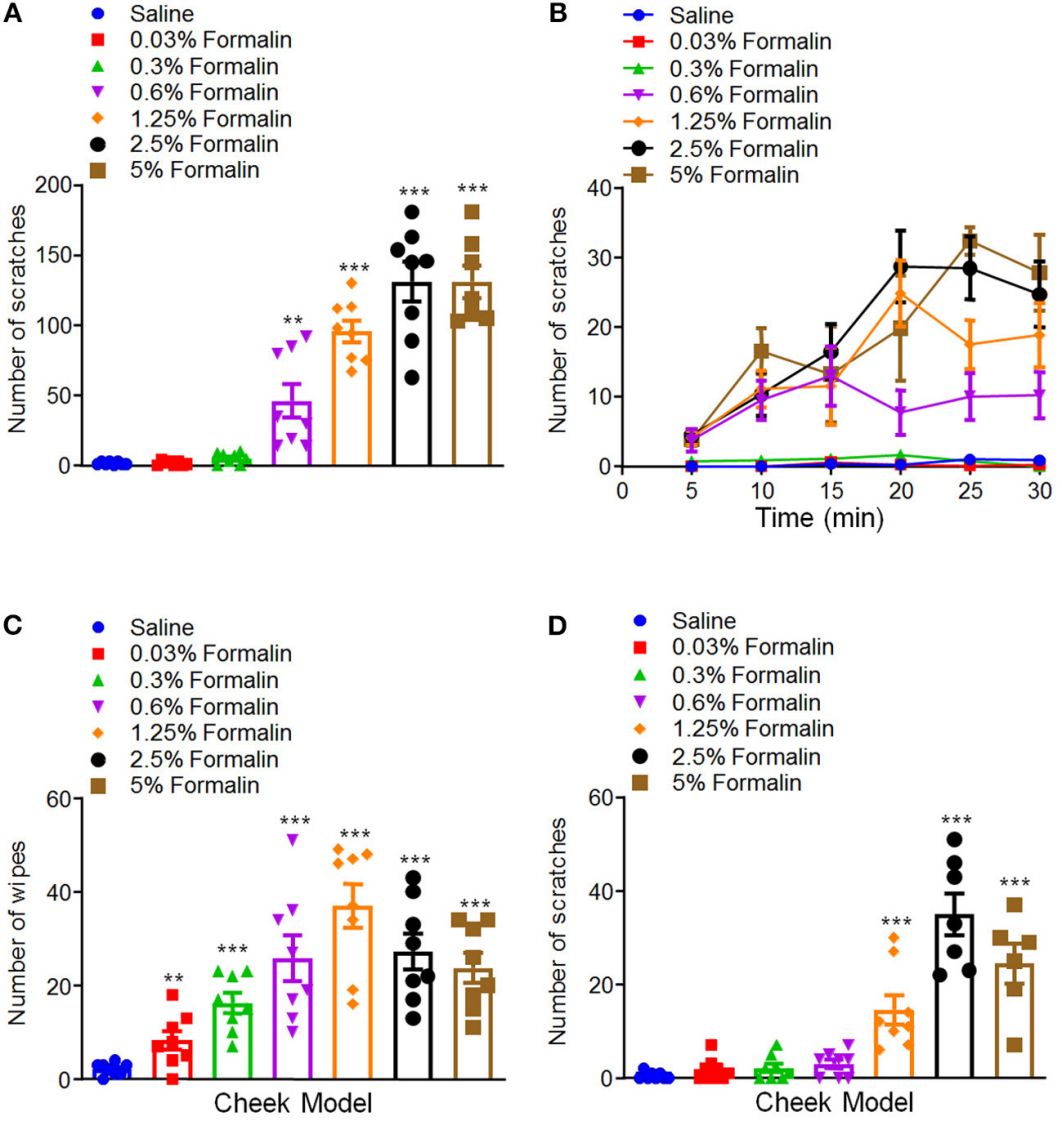
Low-dose formalin evoked wiping behavior in cheek model of rats. **(A,B)** The total number **(A)** and time course **(B)** of scratching behavior induced by i.d. injection of the different doses of formalin (0.03–5%) in the nape of the neck in rats. **(C,D)** The total number of wiping **(C)** and scratching behavior **(D)** induced by i.d. injection of the different doses of formalin (0.03–5%) in the cheek in rats (**P < 0.01, ***P < 0.001 vs. Saline, one-way AVOVA following Bonferroni's test; *n* = 6–8). All data are expressed by means ± SEM.

We further compared the differences of formalin-induced scratching behavior between mice and rats, in order to see whether there are species differences or not. In rats, we found that i.d. injection of formalin (0.03–5%) in the nape of the neck also similarly induced scratching behavior in a dose-dependent manner [*F*_(6, 48)_ = 46.91, *P* < 0.0001; Figures 2A,B]. However, in sharp contrast, the dose–response curve of formalin-induced scratching in rats did not show an inverted “U” shape. In contrast, in the cheek model, we found that low-dose formalin (0.03–0.6%) only induced pain-indicative wiping behavior, but not itch-indicative scratching behavior in rats (Figures 2C,D). Furthermore, higher doses of formalin (1.25–5%) induce mixed wiping [*F*_(6, 49)_ = 12.64, *P* < 0.0001; Figure 2C] and scratching [*F*_(6, 46)_ = 29.34, *P* < 0.0001; Figure 2D] in rats. Together, these data indicated that low-dose formalin induced itch in mice. In contrast, low-dose formalin induced pain in rats, but not in mice. Thus, it indicated that there are significant species differences for low-dose formalin-induced itch behavior.

### Involvement of Opioid Receptors in Low-Dose Formalin-Induced Itch in Mice

**Figure 3:**
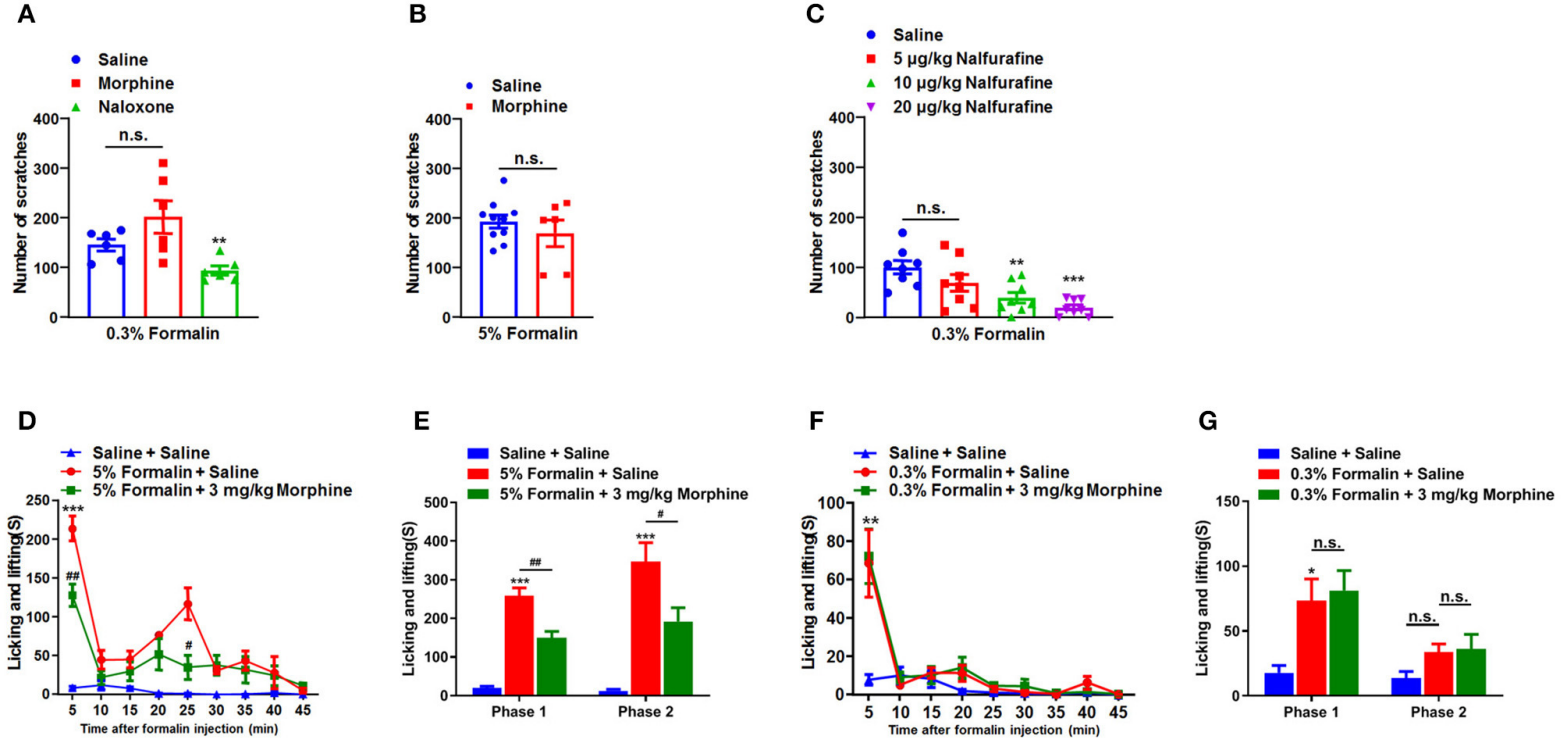
Opioid receptors were involved in the low-dose formalin-induced itch in mice. **(A)** The effects of systemic administration of morphine (1 mg/kg) and naloxone (1 mg/kg), on scratching behavior induced by 0.3% formalin in mice. **(B)** The effects of morphine (1 mg/kg) on 5% formalin-induced itch in mice (*P < 0.05, **P < 0.01, ***P < 0.001 vs. Saline, unpaired Student's *t*-test; *n* = 6–10). **(C)** The effects of systemic administration of nalfurafine on scratching behavior induced by 0.3% formalin in mice (*P < 0.05, **P < 0.01,***P < 0.001 vs. Saline, one-way AVOVA following Bonferroni's test; *n* = 8). **(D,E)** The time course **(D)** and total time **(E)** in nociceptive responses induced by i.pl. 5% formalin and the effects of intraperitoneal (i.p.) injection of morphine (3 mg/kg) on it (*P < 0.05, **P < 0.01,***P < 0.001 vs. Saline + Saline, ^#^*P* < 0.05, ^##^*P* < 0.01, ^###^*P* < 0.001 vs. 5% Formalin + Saline, two-way ANOVA following *post hoc* Bonferroni test; *n* = 6–9). **(F,G)** The time course **(F)** and total time **(G)** in nociceptive responses induced by i.pl. 0.3% formalin and the effects of i.p. injection of morphine (3 mg/kg) on it (*P < 0.05, **P < 0.01,***P < 0.001 vs. Saline + Saline, ^#^*P* < 0.05, ^##^*P* < 0.01 vs. 5% Formalin + Saline, unpaired Student's *t*-test; *n* = 6–9). All data are expressed by means ± SEM. n.s., not significant.

We subsequently explored that possible role of opioid receptors in low-dose formalin-induced itch in mice. Opioid receptors can be divided into three classes: μ-, κ-, and δ-opioid receptors, which are distributed in the central nervous system (CNS) and peripheral nervous system (PNS) (39). It has been reported that μ-opioid receptor agonists evoke itch, while κ-opioid receptor agonists inhibit itch in both animal models and human (40). Consistently, μ-opioid receptor antagonists inhibit itch, while κ-opioid receptor antagonists evoke itch (41). In the present study, μ-opioid receptors agonist morphine (1 mg/kg) and the μ-opioid receptors antagonist naloxone (1 mg/kg) were administered intraperitoneally (i.p.) 30 min before i.d. injection of 0.3% formalin in mice. The results showed that pre-treatment of morphine was not able to affect 0.3% formalin-induced itch (*t*_10_ = 1.610, *P* = 0.1385), while pre-treatment of naloxone was able to significantly reduce 0.3% formalin-induced itch in the neck model of mice (*t*_10_ = 3.417, *P* = 0.0066; Figure 3A). In addition, pre-treatment of morphine also failed to reduce 5% formalin-induced scratching in mice (*t*_14_ = 0.8992, *P* = 0.3837; Figure 3B). Moreover, nalfurafine, a κ-opioid receptor agonist, significantly reduced 0.3% formalin-induced itch in a dose-dependent manner in mice [*F*_(3, 28)_ = 7.962, *P* = 0.0005; Figure 3C].

To further investigate the effects of morphine on behavioral responses induced by intraplantar (i.pl.) injection of 5 or 0.3% formalin into hindpaw in mice, we recorded the total time of licking, shaking, and biting of the injected hindpaw induced by i.pl. injection of formalin. We found that 5% formalin induced licking, shaking, and biting behaviors in phase 1 (1–10 min; *t*_10_ = 11.45, *P* < 0.0001) and phase 2 (10–45 min; *t*_10_ = 6.718, *P* < 0.0001). In addition, pre-treatment of morphine (3 mg/kg; i.p.) significantly reduced 5% formalin-induced phase 1 (*t*_9_ = 3.612, *P* = 0.0056) and phase 2 (*t*_9_ = 2.433, *P* = 0.0378) nociceptive behaviors in mice (Figures 3D,E). In contrast, pre-treatment of morphine (3 mg/kg; i.p.) did not affect 0.3% formalin-induced licking, shaking, and biting behaviors in mice (Figures 3F,G). Thus, these data indicated i.pl. injection of low-dose formalin-induced responses were insensitive to morphine treatment, suggesting these responses may also be itch-related behaviors.

### Low-Dose Formalin-Induced Itch Is Histamine-Independent in Mice

**Figure 4:**
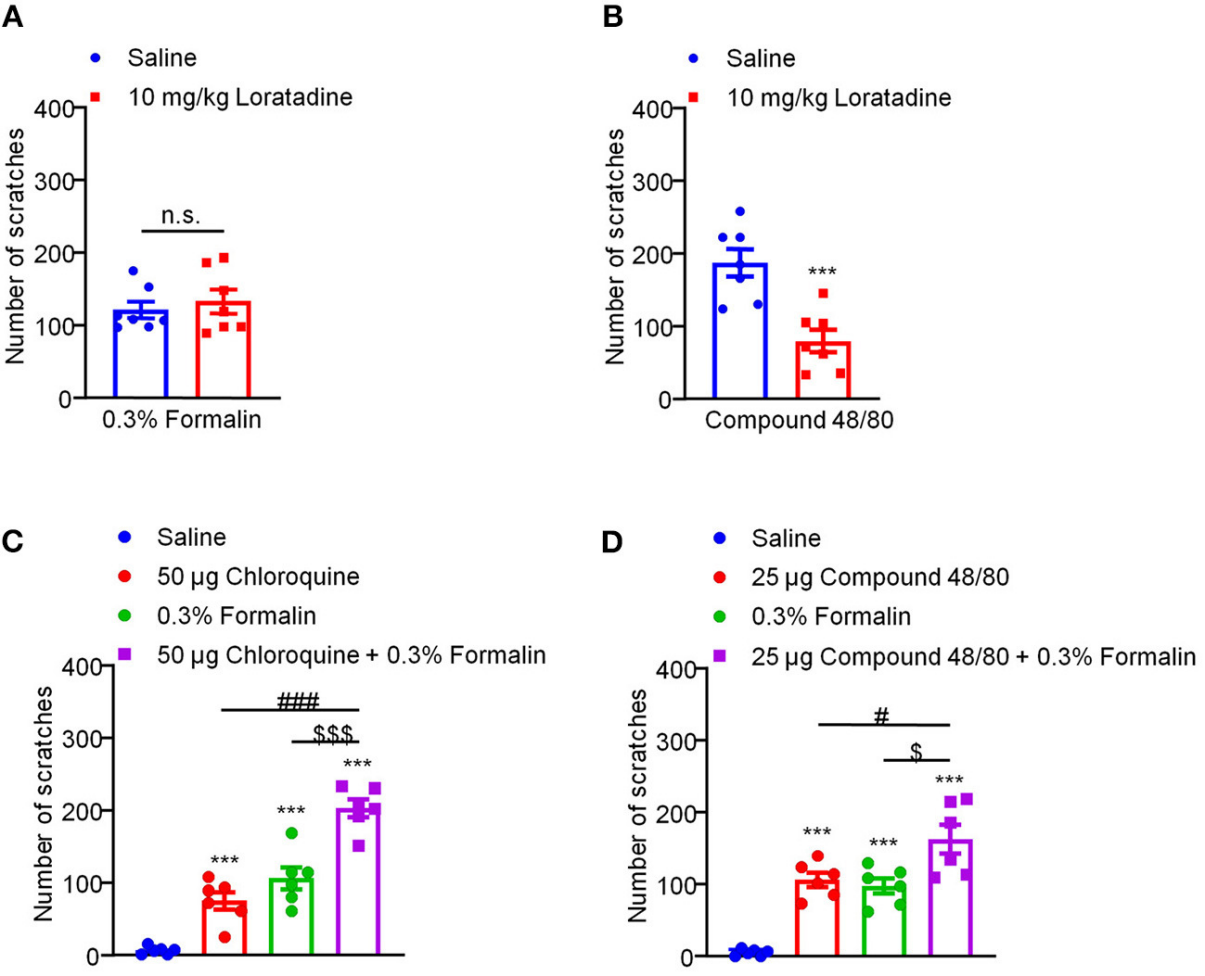
Low-dose formalin-induced itch in a histamine-independent manner in mice. **(A)** The effects of systemic administration of loratadine (10 mg/kg) on 0.3% formalin-induced itch. **(B)** The effects of systemic administration of loratadine (10 mg/kg) on compound 48/80-induced itch (***P < 0.001 vs. Saline, unpaired Student's *t*-test; *n* = 7). **(C)** 0.3% formalin significantly increased chloroquine-induced itch in mice. **(D)** 0.3% formalin significantly increased compound 48/80-induced itch in mice [***P < 0.001 vs. Saline, ^###^*P* < 0.001 vs. 50 μg Chloroquine, ^$$$^*P* < 0.001 vs. 0.3% Formalin, unpaired Student's *t*-test; *n* = 6, **(C)**; ***P < 0.001 vs. Saline, ^#^*P* < 0.001 vs. 25 μg Compound 48/80, ^$^*P* < 0.001 vs. 0.3% Formalin, unpaired Student's *t*-test; *n* = 6, **(D)**]. All data are expressed by means ± SEM. n.s., not significant.

Traditionally, itch is divided into histamine-dependent and histamine-independent itch (42). For example, i.d. injection of compound 48/80 evokes histamine-dependent itch through mast cell degranulation and histamine release (1). CQ, an anti-malarial drug, has been demonstrated to induce histamine-independent itch via activation of Mas-related G protein-coupled receptor A3 (MrgprA3) and TRPA1 in primary sensory neurons in mice (21, 22). Then, we asked whether low-dose formalin-induced itch was histamine-dependent or -independent. Antihistamine loratadine was used as a blocker for histamine H1 receptor (H1R). I.p. injection of loratadine (10 mg/kg) was applied 30 min before i.d. injection of compound 48/80 (100 μg) and 0.3% formalin into the nape of the neck, we found that loratadine failed to inhibit 0.3% formalin-induced itch (*t*_12_ = 0.5733, *P* = 0.5770; Figure 4A). In sharp contrast, loratadine significantly attenuated compound 48/80-induced histamine-dependent itch in mice (*t*_12_ = 4.384, *P* = 0.0009; Figure 4B). In addition, co-administration of 0.3% formalin and CQ (50 μg) significantly increased CQ-induced itch (Saline vs. 50 μg CQ, *t*_10_ = 5.611, *P* = 0.0002; Saline vs. 0.3% Formalin, *t*_10_ = 6.555, *P* < 0.0001; Saline vs. 50 μg CQ + 0.3% Formalin, *t*_10_ = 15.78, *P* < 0.0001; 50 μg CQ vs. 50 μg CQ + 0.3% Formalin, *t*_10_ = 7.458, *P* < 0.0001; 0.3% Formalin vs. 50 μg CQ + 0.3% Formalin, *t*_10_ = 4.999, *P* = 0.0005; Figure 4C). Co-administration of 0.3% formalin and compound 48/80 (25 μg) also significantly increased compound 48/80-induced itch in mice (Saline vs. 25 μg compound 48/80, *t*_10_ = 9.946, *P* < 0.0001; Saline vs. 0.3% Formalin, *t*_10_ = 8.544, *P* < 0.0001; Saline vs. 25 μg compound 48/80 + 0.3% Formalin, *t*_10_ = 7.720, *P* < 0.0001; 25 μg compound 48/80 vs. 25 μg compound 48/80 + 0.3% Formalin, *t*_10_ = 2.490, *P* = 0.0320; 0.3% Formalin vs. 25 μg compound 48/80 + 0.3% Formalin, *t*_10_ = 2.835, *P* = 0.0177; Figure 4D). Therefore, low-dose formalin is able to induce histamine-independent itch in mice.

### TRPA1, but Not TRPV1 or TRPV4, Contributes to Low-Dose Formalin-Induced Itch in Mice

**Figure 5:**
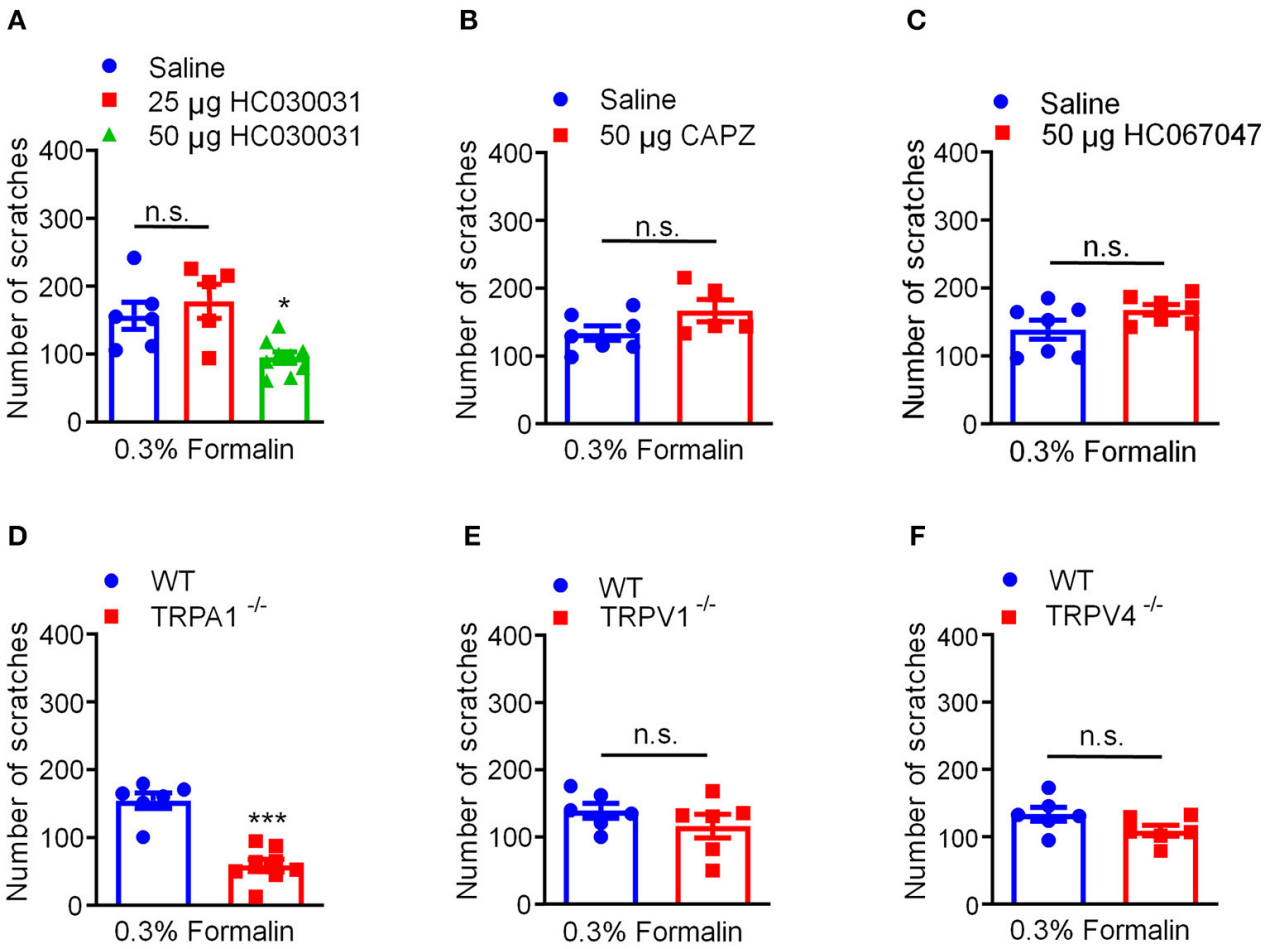
Activation of TRPA1 was required for 0.3% formalin-induced itch in mice. **(A)** The effects of co-administration TRPA1 blocker HC030031 on 0.3% formalin-induced itch in mice. **(B)** The effects of co-administration TRPV1 blocker capsazepine on 0.3% formalin-induced itch in mice. **(C)** The effects of co-administration TRPV4 blocker HC067047 on 0.3% formalin-induced itch in mice. **(D–F)** 0.3% formalin-induced acute itch in mice was abolished in *Trpa1*^−/−^ mice (D), but neither in *Trpv1*^−/−^ mice **(E)** nor in *Trpv4*^−/−^ mice **(F)** [*P < 0.05, ***P < 0.001 vs. 0.3% Formalin, one-way AVOVA following Bonferroni's test; *n* = 5–9, A; *P < 0.05, **P < 0.01, ***P < 0.001 vs. 0.3% Formalin, WT, unpaired Student's *t*-test; *n* = 5–9, **(B–F)**]. All data are expressed by means ± SEM. n.s., not significant.

Previous extensive studies have provided evidence showing TRP ion channels as molecular sensors for chemical, thermal, mechanical, painful, and/or itchy stimuli (43). Previous reports demonstrated that formalin is able to directly activate several TRP channels, including TRPA1 (15), TRPV1 (16), and TRPV4 (17). Then, we explored whether and which TRP ion channels participate in 0.3% formalin-induced itch in mice. We found that co-administration of TRPA1 blockers HC030031 (50 μg) significantly reduced 0.3% formalin-evoked acute itch in mice [*F*_(2, 17)_ = 7.565, *P* = 0.0045; Figure 5A]. However, co-administration of TRPV1 blockers capsazepine (50 μg; *t*_10_ = 1.787, *P* = 0.1043; Figure 5B) or TRPV4 blockers HC067047 (50 μg; *t*_12_ = 1.824, *P* = 0.0932; Figure 5C) failed to affect 0.3% formalin-evoked itch in mice. Consistently, 0.3% formalin-induced acute itch in mice was abolished in *Trpa1*^−/−^ mice compared with WT mice (*t*_12_ = 6.630, *P* < 0.0001; Figure 5D). In sharp contrast, 0.3% formalin-evoked acute itch was affected in neither *Trpv1*^−/−^ mice (*t*_10_ = 1.087, *P* = 0.3026; Figure 5E) nor *Trpv4*^−/−^ mice (*t*_10_ = 1.819, *P* = 0.0989; Figure 5F). Therefore, activation of TRPA1 (but not TRPV1 or TRPV4) is required for low-dose formalin-induced acute itch.

### Oxidative Stress Contributes to Low-Dose Formalin-Induced Itch in Mice

**Figure 6:**
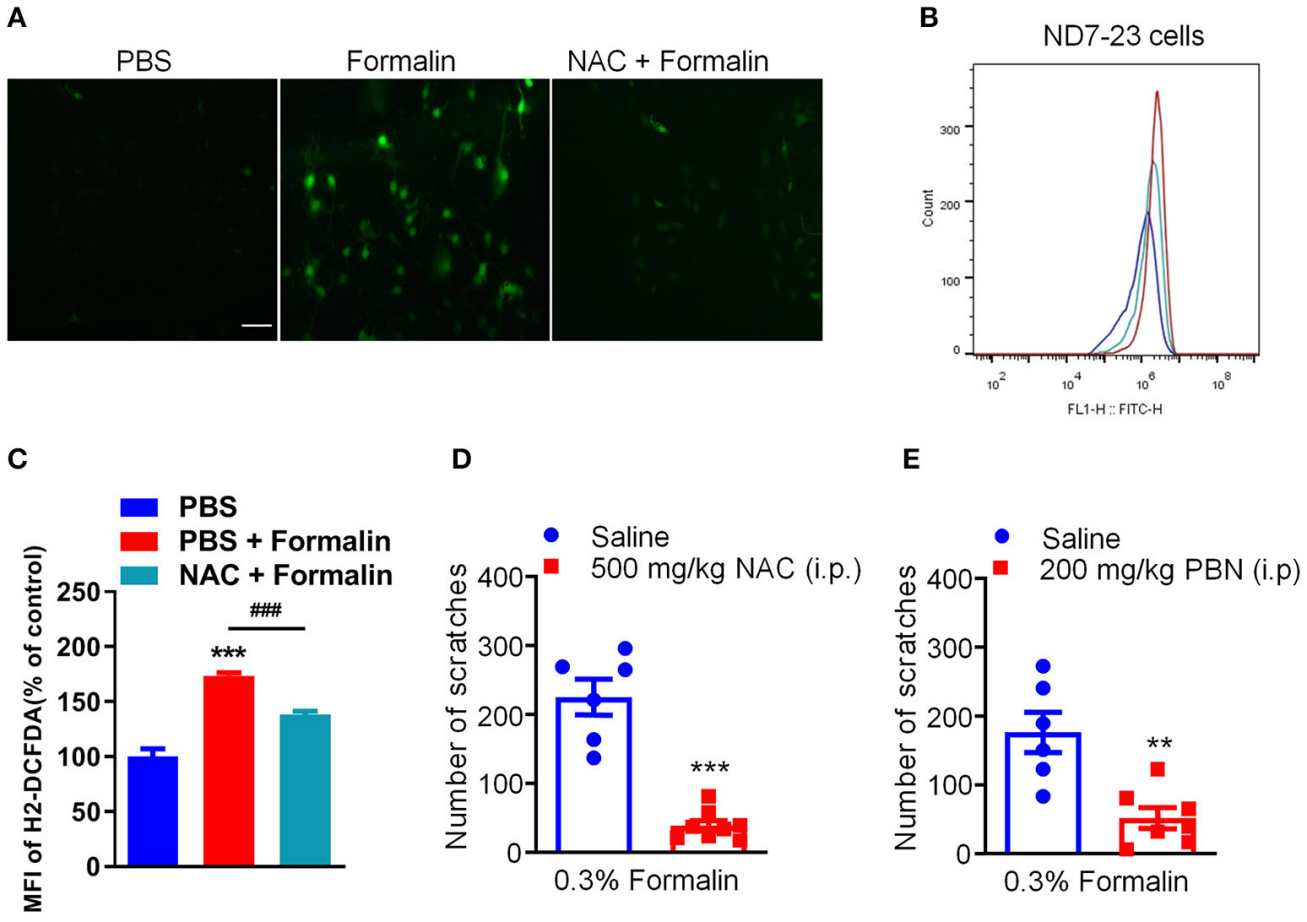
The effects of antioxidants on low-dose formalin-induced itch. **(A)** Representative fluorescence images of intracellular ROS stained with DCFH-DA probe showing that 0.03% formalin-induced significant accumulation of intracellular ROS, which was suppressed by the antioxidants NAC. **(B,C)** Flow cytometry **(B)** and quantification **(C)** confirmed that incubation with 0.03% formalin increased intracellular ROS, which was inhibited by the antioxidants NAC (**P < 0.01; ***P < 0.001 vs. PBS, ^###^*P* < 0.001 vs. PBS + Formalin, unpaired Student's *t*-test; *n* = 3). **(D,E)** The effects of systemic administration of NAC (500 mg/kg) and PBN (200 mg/kg) on 0.3% formalin-induced itch in mice [**P < 0.01, ***P < 0.001 vs. Saline, unpaired Student's *t*-test; *n* = 6–8, **(D,E)**]. All data are expressed by means ± SEM. n.s., not significant.

Our previous studies have demonstrated that oxidative stress plays a critical role in the genesis of histamine-independent itch (23, 24). In the present study, we investigated whether 0.3% formalin directly increases the level of intracellular ROS in the ND7-23 cells, a DRG-derived cell line (23). The intracellular ROS generation and scavenging were measured using DCFH-DA, a fluorescent probe for the highly-selective detection of superoxide in live cells (23). We found that incubation with 0.03% formalin significantly increased intracellular ROS in the ND7-23 cells, as reflected by enhanced DCFH-DA fluorescence intensity compared with control, while antioxidant NAC remarkably decreased it (Figure 6A). Moreover, we used flow cytometry to quantify intracellular ROS generation (Figures 6B,C). When ND7-23 cells were exposed to 0.03% formalin, the mean fluorescence intensity (MFI) was higher than with vehicle treatment, and this was attenuated by pre-treatment with NAC (PBS vs. PBS + Formalin, *t*_4_ = 9.709, *P* = 0.0006, PBS + Formalin vs. NAC + Formalin, *t*_4_ = 9.373, *P* = 0.0007; Figure 6C). Thus, our results demonstrated that 0.03% formalin directly caused the accumulation of intracellular ROS in ND7-23 cells and antioxidants attenuated it. Furthermore, two commonly used antioxidants, NAC and PBN, were i.p. administered 30 min before i.d. injection of 0.3% formalin into the nape of the neck in mice. The results showed that low-dose formalin-induced scratching was significantly reduced by pre-treatment with NAC (*t*_12_ = 7.817, *P* < 0.0001; Figure 6D) or PBN (*t*_11_ = 3.938, *P* = 0.0023; Figure 6E) in mice.

### Activation of p-ERK Signaling in the Dorsal Root Ganglion Contributes to Low-Dose Formalin-Induced Itch in Mice

**Figure 7:**
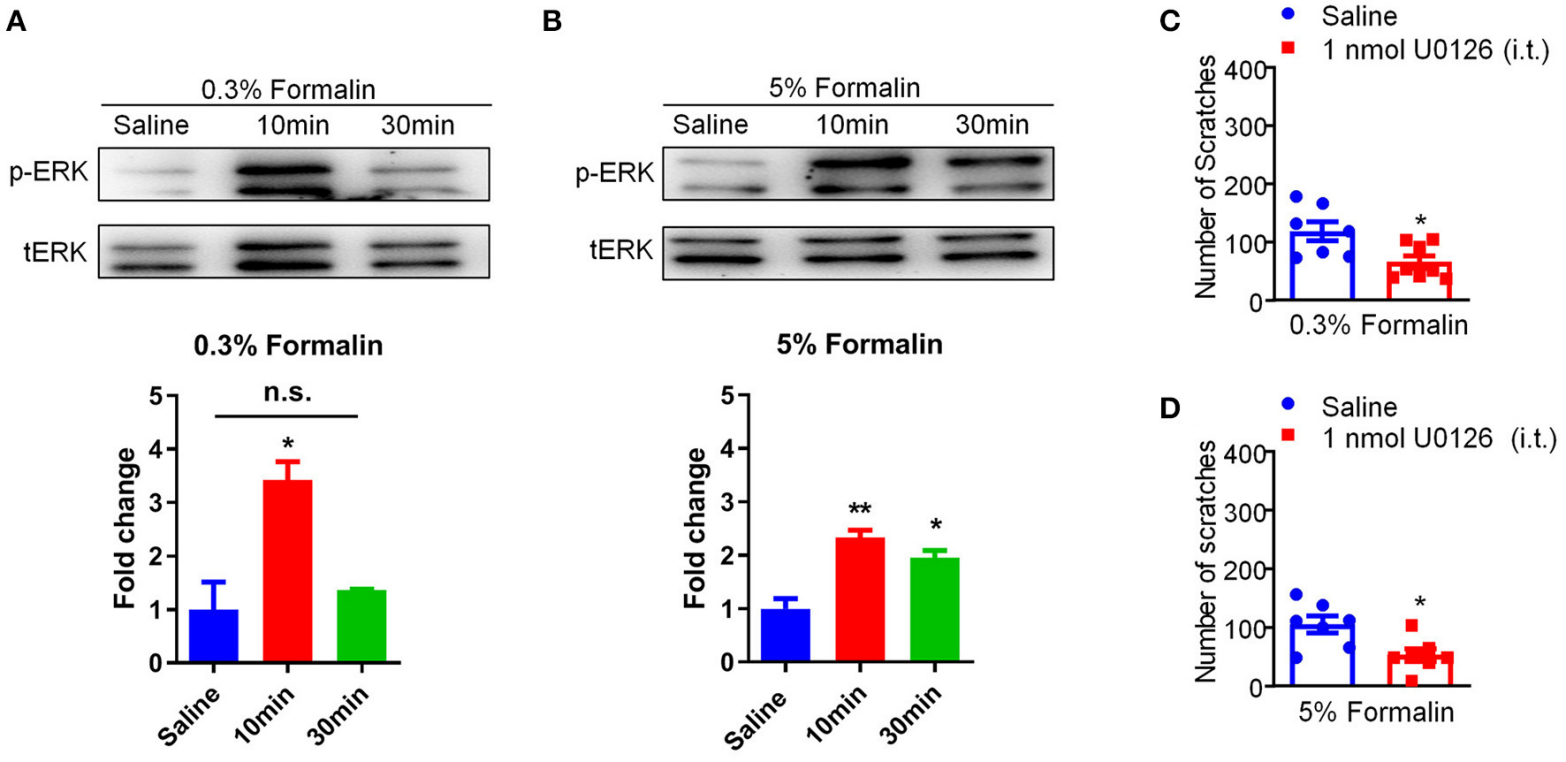
Activation of p-ERK in the DRGs was involved in low-dose formalin-induced itch in mice. **(A,B)** Western blots (upper panel) and quantification (lower panel) showing that p-ERK expression was significantly increased in 10 min and 30 min in the DRG after i.d. injection of 0.3% **(A)** and 5% formalin **(B)** (*P < 0.05, **P < 0.01 vs. Saline, unpaired Student's *t*-test; *n* = 3). **(C,D)** Intrathecal (i.t.) injection of U0126 (1 nmol) decreased both 0.3% **(C)** and 5% formalin **(D)**-induced scratching behavior in mice (*P < 0.05, **P < 0.01 vs. Saline, unpaired Student's *t*-test; *n* = 7–8). All data are expressed by means ± SEM. n.s., not significant.

Previous reports demonstrated that phosphorylation of extracellular signal-regulated kinase (ERK) in the DRG and spinal cord contributes to the genesis of both pain (44) and itch (45). In our study, we confirmed that i.d. injection of 0.3% formalin (Saline vs. 0.3% Formalin 10 min, *t*_3_ = 3.898, *P* = 0.0107) and 5% formalin (Saline vs. 5% Formalin 10 min, *t*_4_ = 5.722, *P* = 0.0046; Saline vs. 5% Formalin 30 min, *t*_4_ = 4.074, *P* = 0.0152) up-regulated p-ERK in the DRG in 10 and 30 min, respectively (Figures 7A,B). Moreover, intrathecal (i.t.) injection of the mitogen-activated protein kinase (MEK) inhibitor U0126 (1 nmol) inhibited both low-dose (*t*_13_ = 2.808, *P* = 0.0148; Figure 7C) and high-dose (*t*_12_ = 2.922, *P* = 0.0128; Figure 7D) formalin-induced acute itch in mice. Thus, our results demonstrated that p-ERK activation in the DRG was involved in formalin-induced acute itch in mice.

### Incubation of Low-Dose Formalin Increases Intracellular Calcium in Primary Cultured Dorsal Root Ganglion Neurons From Mice

**Figure 8:**
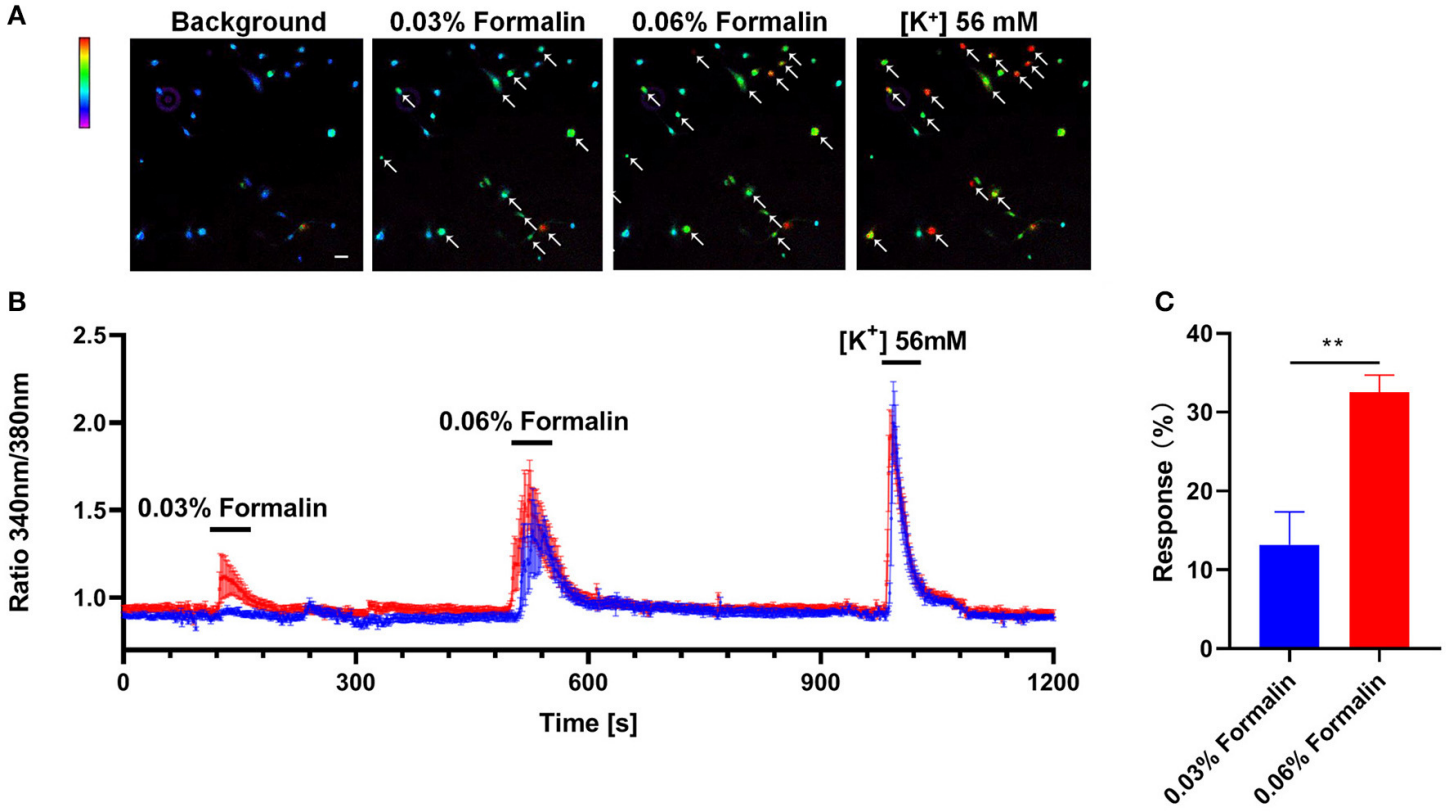
Direct activation of DRG neurons by low-dose formalin. Ca^2+^ imaging of primary cultured DRG neurons with the indicator Fluo-2 AM **(A)** Representative Fura-2 fluorescence heat map images of DRG neurons before and after application of 0.03% formalin, 0.06% formalin, and 56 mM KCl (scale bar, 100 μm). **(B)**. Representative traces of intracellular Ca^2+^ responses induced by application of 0.03% formalin, 0.06% formalin, and 56 mM KCl (blue traces, *n* = 5; red traces, *n* = 7). **(C)** The response (%) of primary DRG neurons with 0.03% and 0.06% formalin and 56 mM KCl treatment. All data are expressed by means ± SEM. n.s., not significant (**P < 0.01 vs. 0.03% formalin, unpaired Student's *t*-test; *n* = 4).

We further explored the direct activation of the primary cultured DRG neurons with different doses of formalin by calcium imaging experiments. We found that a subset of DRG neurons (about 13.17 ± 3.61%) could be activated by low-dose formalin (0.03%), while significantly more neurons (32.52 ± 1.91%) could be activated by high-dose formalin (0.06%) (*P* < 0.01; Figures 8A–C). Thus, these results indicate that low-dose formalin activates a small subset of neurons, while higher-dose formalin activates a larger population of DRG neurons.

## Discussion

Itch is a distinct sensory modality of the somatosensory system of mammals. Acute itch is considered as a protective mechanism to remove potentially harmful stimuli. However, chronic itch is a clinical challenging problem in many dermatological or systemic diseases. To elucidate the mechanisms of itch and to screen new anti-itch compounds, reliable pre-clinical itch animal models are urgently needed to be developed. In the present study, we established an acute histamine-independent itch mouse model by i.d. injection of low-dose formalin in the neck or the cheek. It was found that 0.3% formalin induced histamine-independent itch by activation of TRPA1 in mice, but not in rats. Furthermore, 0.3% formalin-induced itch was inhibited by κ-receptor agonist, antioxidants, and MEK inhibitor. Thus, these data supported the idea that this new “formalin itch test” may be useful for the screening of novel anti-itch drugs.

### Low-Dose Formalin Induces Itch in Mice: A New Acute Itch Model?

To date, there are several acute itch models that were developed for studying underlying mechanisms or screening anti-itch drugs, including acute itch induced by histamine (46), compound 48/80 (47), CQ (21), endothelin-1 (48), 5-HT (49), H_2_O_2_ (23), and imiquimod (31). Given different pruritogens may induce itch through a distinct mechanism, different acute itch models are still needed to be developed. Since first reported over 40 years ago, the formalin test has been widely used in pain research and evaluation of analgesic drugs in laboratory animals (50), and formalin test is known to capture some mechanisms that are likely to be relevant to many pain patients in clinic (51). The nociceptive responses induced by formalin are marked by licking, biting, lifting, and shaking the injected hindpaw in rodents (52). The formalin test is well-known with a biphasic (early and late) nociceptive response in rodent (53). The early phase (phase 1) is characterized by acute peripheral activation of C and Aδ fibers, while the late phase (phase 2) involves persistent inflammatory nociceptive inputs and the development of central sensitization (54). However, whether and how formalin induces itch are unclear. In a previous report, subcutaneous (s.c.) injections of pruritogenic agents, such as compound 48/80 and substance P, in the rostral back induced scratching in mice (50). In contrast, s.c. injection of algesiogenic agents, such as capsaicin (30 and 100 μg) and dilute formalin (5 mg formaldehyde), into the rostral back was without significant effects in mice (55). Thus, they concluded that pruritogenic (but not algesiogenic) agents are able to induce scratching behavior in mice, and scratching behavior was considered to be a reliable measurement for itch testing (55). Interestingly, there were several studies that suggested that i.d. injection of formalin may be able to evoke itch-associated scratching in mice (34, 35). In the present study, we demonstrated that i.d. injection of the different doses of formalin (0.3–5%) in mice can evoke scratching behavior in a dose-dependent manner. The dose–response curve of formalin-induced scratching showed an inverted “U” shape, which was consistent with our previous reports (7, 23). Further, the cheek model showed that the low-dose formalin (0.3–0.6%) only induced itch-indicative scratching; however, the higher doses of formalin (1.25–5%) induce mixed itch and pain in mice. Low-dose formalin-induced itch was significantly inhibited by κ-receptor agonist nalfurafine, but not μ-opioid receptor agonist morphine. After i.pl. injection of formalin, we found that both 0.3% formalin and 5% formalin induced acute pain in phase 1, and 5% formalin evoked inflammatory pain in phase 2. In addition, systemic application of morphine reduced i.pl. injection of 5% formalin-induced acute pain and inflammatory pain, while it failed to reduce 0.3% formalin-evoked responses in mice. Thus, we provided strong behavioral and pharmacological evidence to support that low-dose formalin induced itch behaviors in mice.

Furthermore, we also found that i.d. injection of the different doses of formalin into the nape of the neck induced scratching behavior in a dose-dependent manner in rats without an inverted “U” shape for the dose–response curve. In sharp contrast, the cheek model indicated that low-dose formalin only induced pain-indicative wiping, but the higher doses of formalin (1.25–5%) induced mixed itch and pain in rats. Thus, although higher-dose formalin induced mixed itch and pain in both mice and rats, low-dose formalin induced only itch in mice, but not in rats. Although formalin test in both mice and rats was widely used for pain research, these results indicated that there are significant species differences for low-dose formalin-induced itch between mice and rats.

### TRPA1 Mediates Low-Dose Formalin-Induced Itch in Mice

We asked which TRP channels are involved in low-dose formalin-induced itch in mice. First, we found that systemic administration of the histamine H1R blocker loratadine fails to inhibit 0.3% formalin-induced itch, indicating 0.3% formalin-induced itch in a histamine-independent manner. Co-administration of TRPA1 blockers HC030031 significantly reduced 0.3% formalin-evoked acute itch in mice, but not for TRPV1 and TRPV4 inhibitors. In addition, 0.3% formalin-induced acute itch was abolished in *Trpa1*^−/−^ mice, but not affected in *Trpv1*^−/−^ and *Trpv4*^−/−^ mice. Thus, these data indicated that activation of TRPA1 is required for low-dose formalin-induced itch in mice. Although previous studies demonstrated that formalin is able to activate several TRP channels, including TRPV1 (16), TRPA1 (15), and TRPV4 (17), our results indicated that only TRPA1 activation contributes to low-dose formalin-induced itch in mice, indicating that different TRP channels mediate different behaviors induced by formalin. Our results also emphasized that TRPA1 acts as receptor for many pruritogens, including H_2_O_2_ (23), methylglyoxal (7), imiquimod (30), and low-dose formalin in this study. Recently, it was reported that a non-covalent agonist (GNE551) had distinct binding pocket and ligand-interaction mechanism for TRPA1 (56). Unlike the covalent agonist AITC, GNE551 activated TRPA1 without desensitization and induced persistent pain (51). Thus, targeting TRPA1 may be a novel strategy for developing new anti-nociception or anti-itch compounds, which is consistent with previous reports (7, 22, 23).

### The Roles of Oxidative Stress and p-ERK Signaling in Low-Dose Formalin-Induced Itch

Previous reports have shown that oxidative stress plays a key role in the pathogenesis of acute and chronic itch (23, 24). In the present study, we found that accumulation of intracellular ROS induced by 0.03% formalin in cultured DRG-derived cell line ND7-23, which was suppressed by perfusion of antioxidant NAC. In line with this observation, incubation of compound 48/80 or CQ also significantly increased the level of intracellular ROS in ND7-23 cells (24). Thus, these data indicated that intracellular ROS may act as second messengers for itch signaling transduction. In addition, systemic administration of the antioxidants NAC and PBN markedly reduced low-dose formalin-induced itch in mice. Thus, it indicated that oxidative stress in the DRGs is involved in low-dose formalin-induced itch and antioxidants may act as promising anti-itch compounds.

Previous work suggested that p-ERK activation in the DRGs and the spinal cord is involved in the genesis of itch (24, 45). In the present work, it was found that i.d. injection of 0.3% formalin transiently up-regulated p-ERK in the DRGs, while 5% formalin persistently up-regulated p-ERK in the DRG. Moreover, i.t. injection of the MEK inhibitor U0126 inhibited both low-dose and high-dose formalin-induced itch behaviors in mice. Thus, p-ERK up-regulation in the DRG was involved in low-dose formalin-induced itch in mice.

Finally, we found that the primary DRG neurons could be directly activated by low-dose formalin by using calcium imaging analysis. The results showed that a subset of DRG neurons are sensitive to low-dose formalin, while a subpopulation of DRG neurons are responsive to both low-dose (0.03%) and high-dose (0.06%) formalin. The results indicate that low-dose formalin may directly activate a subset of DRG neurons, which may be selectively involved in itch signaling transduction. However, the identity, function, and sex difference of this subpopulation of DRG neurons that are sensitive to low-dose formalin remain to be investigated.

### Is Formaldehyde a Novel Pruritogen?

Our study has developed a novel acute histamine-independent itch model, which may be useful for itch mechanistic studies and screening for anti-itch drugs. Furthermore, I wondered whether there is clinical relevance for low-dose formalin-induced itch or not. A previous study showed that exposure to formaldehyde aggravated pruritus and skin inflammation in a rat model of atopic dermatitis (57). In addition, many patients undergoing chronic hemodialysis are suffering from chronic itch (9). Interestingly, these patients are often exposed to formaldehyde (58), although the causal relationship between uremic pruritus and formaldehyde is unclear. For atopic dermatitis, exposure to formaldehyde causes skin barrier dysfunction in patients, suggesting that formaldehyde may exacerbate atopic dermatitis (59). Repeated exposure of formaldehyde can cause allergic contact dermatitis in both human (60) and animal models (61). Given many cytokines (especially IL-31) contribute to the pathogenesis of chronic itch, the roles of cytokines and/or chemokines in chronic itch induced by repeated exposure formaldehyde warrant further investigation. Intriguingly, endogenous formaldehyde can also be produced in the body, especially under pathological conditions, such as cancer (62) and Alzheimer's disease (63). The roles of endogenous formaldehyde in chronic itch remain unclear.

In summary, we have provided strong evidence that the low-dose formalin is able to induce histamine-independent itch in mice (but not in rats), which is mediated by the activation of the TRPA1 channel. Thus, we have developed a new pre-clinical itch animal model, which may be helpful to pruritus research.

## Data Availability Statement

The raw data supporting the conclusions of this article will be made available by the authors, without undue reservation.

## Ethics Statement

The animal study was reviewed and approved by the Ethics Committee for the Use of Experimental Animals in Soochow University Animal Committee.

## Author Contributions

XL, J-TZ, YH, W-QS, Z-HW, Q-YF, D-NF, JJ, and TL contributed to the work design, performed experiments, and analyzed and interpreted data from all the experiments. XL, J-TZ, YH, W-QS, Z-HW, Q-YF, and D-NF carried out *in vivo* animal experiments. J-TZ, YH, W-QS, and Q-YF performed *in vitro* experiments. J-TZ, YH, JJ, and TL prepared and finalized the manuscript. All authors have critically revised and approved the final manuscript and agreed to be accountable for all aspects of the work.

## Conflict of Interest

The authors declare that the research was conducted in the absence of any commercial or financial relationships that could be construed as a potential conflict of interest.
